# Improving Emergency Department Patient-Physician Conversation Through an Artificial Intelligence Symptom-Taking Tool: Mixed Methods Pilot Observational Study

**DOI:** 10.2196/28199

**Published:** 2022-02-07

**Authors:** Justus Scheder-Bieschin, Bibiana Blümke, Erwin de Buijzer, Fabienne Cotte, Fabian Echterdiek, Júlia Nacsa, Marta Ondresik, Matthias Ott, Gregor Paul, Tobias Schilling, Anne Schmitt, Paul Wicks, Stephen Gilbert

**Affiliations:** 1 Department of Interdisciplinary Acute, Emergency and Intensive Care Medicine (DIANI), Klinikum Stuttgart Stuttgart Germany; 2 Ada Health Berlin Germany; 3 Department of Nephrology, Klinikum Stuttgart Stuttgart Germany; 4 Department of Infectious Diseases, Klinikum Stuttgart Stuttgart Germany; 5 The Else Kröner Fresenius Center for Digital Health University Hospital Carl Gustav Carus Dresden Technische Universität Dresden Dresden Germany

**Keywords:** symptom assessment application, anamnesis, health care system, patient history taking, diagnosis, emergency department

## Abstract

**Background:**

Establishing rapport and empathy between patients and their health care provider is important but challenging in the context of a busy and crowded emergency department (ED).

**Objective:**

We explore the hypotheses that rapport building, documentation, and time efficiency might be improved in the ED by providing patients a digital tool that uses Bayesian reasoning–based techniques to gather relevant symptoms and history for handover to clinicians.

**Methods:**

A 2-phase pilot evaluation was carried out in the ED of a German tertiary referral and major trauma hospital that treats an average of 120 patients daily. Phase 1 observations guided iterative improvement of the digital tool, which was then further evaluated in phase 2. All patients who were willing and able to provide consent were invited to participate, excluding those with severe injury or illness requiring immediate treatment, with traumatic injury, incapable of completing a health assessment, and aged <18 years. Over an 18-day period with 1699 patients presenting to the ED, 815 (47.96%) were eligible based on triage level. With available recruitment staff, 135 were approached, of whom 81 (60%) were included in the study. In a mixed methods evaluation, patients entered information into the tool, accessed by clinicians through a dashboard. All users completed evaluation Likert-scale questionnaires rating the tool’s performance. The feasibility of a larger trial was evaluated through rates of recruitment and questionnaire completion.

**Results:**

Respondents strongly endorsed the tool for facilitating conversation (61/81, 75% of patients, 57/78, 73% of physician ratings, and 10/10, 100% of nurse ratings). Most nurses judged the tool as potentially time saving, whereas most physicians only agreed for a subset of medical specialties (eg, surgery). Patients reported high usability and understood the tool’s questions. The tool was recommended by most patients (63/81, 78%), in 53% (41/77) of physician ratings, and in 76% (61/80) of nurse ratings. Questionnaire completion rates were 100% (81/81) by patients and 96% (78/81 enrolled patients) by physicians.

**Conclusions:**

This pilot confirmed that a larger study in the setting would be feasible. The tool has clear potential to improve patient–health care provider interaction and could also contribute to ED efficiency savings. Future research and development will extend the range of patients for whom the history-taking tool has clinical utility.

**Trial Registration:**

German Clinical Trials Register DRKS00024115; https://drks.de/drks_web/navigate.do?navigationId=trial.HTML&TRIAL_ID=DRKS00024115

## Introduction

### Background

The emergency department (ED) is, by definition, a high-stress environment. As it is so critical that the time of a health care provider (HCP) is used optimally, effective and empathetic communication with patients (and colleagues) can be challenging [1]. There is increasing recognition that hospital EDs face numerous challenges related to crowding, a problem likely to continue for the foreseeable future [2]. Obvious barriers to effective communication include time pressures caused by a full waiting room and urgent cases exceeding capacity [1]. More subtle and pervasive systemic factors include limitations of processes and interpersonal parameters such as societal and health disparities [3].

It has been proposed that appropriately designed artificial intelligence (AI)–based systems could reduce ED documentation errors, improve patient safety [4-8], and free up HCP time. Such time savings could potentially be used to improve efficiency and provide HCPs with more time to build rapport with patients [5].

One such AI-based digital symptom assessment tool is Ada (Ada Health GmbH), which uses a Bayesian probabilistic reasoning engine with an adaptive question flow to collect demographic information, medical history, and symptoms. Previous studies have demonstrated that the Ada tool was helpful and easy to use for patients in a primary care waiting room [9] and has the potential to reduce waiting times and increase efficiency at urgent care centers [10]. Its underlying technology has the highest condition-suggestion accuracy among tools of its class and has the same quality of safe urgency advice as general practitioners [11]. When integrated at a large US health care system, its urgency-advice accuracy recommendations were stated by independent researchers to be comparable to those of nurse-staffed telephone triage lines [12]. A number of studies [9,13,14] have described a range of tools for self-assessment of urgency and triage in the general practice and acute primary care setting, but studies evaluating the handover of history and symptom information to HCPs have not been carried out with real patients. If appropriately adapted to the ED setting, the benefits of a digital history-taking tool could assist nurse-led triage in the waiting room and assessment and treatment by ED physicians. This study used methodologies adopted in other evaluations of new digital technologies for patient self-reported history in the ED setting [15,16].

### The Aim of This Study

In this study, we aim to evaluate a prototype digital history-taking and handover system, which includes a patient-facing tool for symptom and history taking. After patients inputted their data in the patient-facing tool, a clinical handover report was displayed to the physician or nurse using an HCP-facing tool. Our primary hypothesis was that such a tool might alleviate some of the challenges in building rapport and communication in a crowded ED. The secondary hypotheses were that the tool could alleviate documentation and time pressures. This pilot study, which was based on the approach reported in some studies [16-18], assessed in 2 study stages a prototype patient-facing system to assist communication. First, phase 1 involved initial implementation of the patient-facing and HCP-facing tools (version V1), followed by their evaluation by all users. Feedback on performance from phase 1 was then used to create a modified system (version V2), which was further evaluated in phase 2. Patients, physicians, and nurses quantitatively evaluated the 2 system versions in terms of their usability and usefulness in facilitating patient-HCP conversation and rapport formation in the ED setting. For HCP users, we also explored the helpfulness of the medical information provided at handover and its perceived potential for the system to save HCP time.

## Methods

### Design: Overall Study Approach and Study Type

This study used incremental mixed methods approaches and was designed such that phase 1 allowed initial learning about the tool [16-18] and co-design between researchers and HCPs. Modifications suggested by users that could be implemented within the project time frame were then used to develop a V2 prototype, and at a switchover point, the V1 tool was replaced by the V2 tool, which then remained unchanged during phase 2. There were no study design changes made between phase 1 and phase 2. The study was conducted between August 3, 2020, and August 21, 2020.

This study explored the potential for enhancing bidirectional patient-HCP communication in the ED through the introduction of an augmentative prototype history and symptom-taking tool. Questionnaire-based user perceptions on the potential for the system were collected alongside qualitative observations. The mixed methods approach, along with the full implementation and use of the tool for patients, provided real-world data in a manner often only achievable through interventional study designs. However, the overall study approach was observational because the system was not closely integrated into HCP clinical workflows during the study and did not replace standard practice in the ED. HCPs were carefully trained not to rely on the prototype system for formal or definitive symptom-taking support during the study. In this way, no interventional patient outcome measures were recorded. This study was conducted in accordance with requirements of the Standards for Quality Improvement Reporting Excellence [19], SPIRIT-AI (Standard Protocol Items: Recommendations for Interventional Trials–Artificial Intelligence), and CONSORT-AI (Consolidated Standards of Reporting Trials–Artificial Intelligence) [20] guidelines.

### Description of the Prototype Digital Symptom and History-Taking System

**Figure 1:**
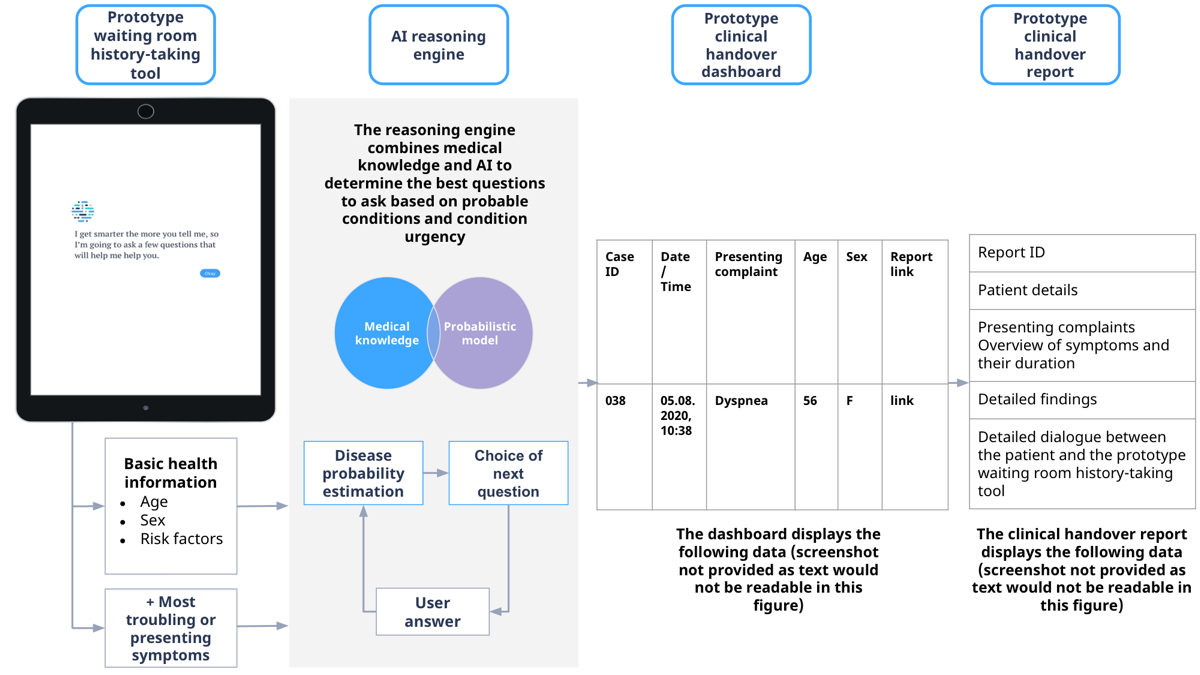
The prototype digital history and symptom-taking and handover system evaluated in this study showing the interactions between the patient-facing and health care provider–facing tools and describing how the artificial intelligence (AI) reasoning engine functions to sequentially ask the patient the most relevant question next. Although the screenshots presented are in English, the tool used in this study was in German.

The system consisted of patient- and HCP-facing tools (Figure 1). The patient-facing tool was provided to recruited patients in the waiting room on a tablet computer (iPad, Apple Inc). The patient history–taking tool used a cloud-based Bayesian probabilistic reasoning engine combined with curated medical knowledge to ask the patient the optimal set of questions based on probability and urgency of conditions. The Ada reasoning engine versions used in the study were version 1.31.1 and version 1.31.2. The assessment was performed as an interaction-enabled question flow with options to confirm, deny, or skip each question. The tool asked successively tailored questions about the respondent’s medical history and the main presenting complaints, as well as related attributes of their symptoms, such as severity and time course.

The HCP-facing tool provided a secure web-interface dashboard that listed all completed assessments to the ED clinical staff. The tool also provided a detailed handover report, designed to provide clinical information quickly and safely to HCPs. The handover report included the patient’s basic information (sex and year of birth); basic medical history information (smoker, hypertension, diabetes, and pregnancy status); main presenting symptoms; details of these symptoms, including the specific questions asked by the tool; and answers provided by the patient.

### Design: Study Procedure

**Figure 2:**
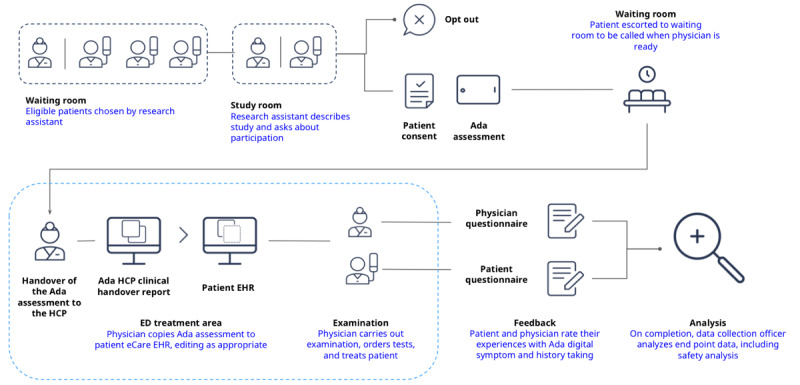
Overview of the study procedure. ED: emergency department; EHR: electronic health record; HCP: health care provider.

The procedure of the study is described in Figure 2.

#### Recruitment, Inclusion, and Exclusion Criteria

The following inclusion criteria were applied: (1) patients aged >18 years, (2) attended the Stuttgart study site when recruitment was being undertaken, and (3) willing and able to provide consent. The exclusion criteria were as follows: (1) patients with severe injury or illness requiring immediate treatment, that is, Emergency Severity Index (ESI) levels 1-2 (patients classified as ESI level 3 were included if assessed as suitable) [21]; (2) patients with traumatic injury; (3) patients incapable of completing a health assessment, for example, because of illiteracy, mental impairment, or inebriation or other incapacity; and (4) patients aged <18 years. The clinician research assistant asked all potentially eligible patients in the waiting room if they would be interested in participating in a study evaluating a tool to record and pass on their symptoms to the ED physicians. Potentially eligible patients were informed that the study would not delay (or accelerate) their treatment at the ED. A single clinician research assistant carried out enrollment and consent.

#### Informed Consent and Study Data Management

If the patient agreed to be considered for inclusion, they were led to a separate private room adjacent to the ED where the nature, background, and scope of the study were explained and they were asked if they wanted to participate. If the patient consented, the pseudoanonymization procedure was followed. The patient’s name was recorded alongside the next-in-sequence study patient enrollment number on the study enrollment (disambiguation) record, which is the only link between the study ID (study patient enrollment number) and patient name, which was kept securely by the principal investigator (TS). All quantitative and qualitative data were only accessible by the study team on secure systems. The study questionnaire data were recorded and stored at the study site in secure databases. Paper surveys were stored in secure hospital clinical trials file storage. The patient’s enrollment number was entered into the patient-facing tool on the iPad, and the tool then asked the patient to agree to the terms and conditions and privacy policy. The study team was familiar with data privacy regulations and is committed to data protection principles. The study was approved by the local ethics committee at the University of Heidelberg (S-052-2020) and is registered in the German Clinical Trials Register (DRKS00024115).

#### Procedure for Patient Symptom Taking

The clinician research assistant answered any questions the patient had about the tool and helped them to use it if requested, recording the degree of help provided. The symptom-taking handover report was not provided to the patient and was automatically made available to the ED physicians in the ED treatment area through the HCP-facing tool.

#### Training and Study Procedure for HCPs in the ED

All the ED nurses and physicians were made aware of the study in advance through a presentation of the study and a written manual describing the system. Physicians were made aware of all patients who were enrolled in the study. The HCP logged onto the secure web interface using a secure ID to access the handover report.

### Study Measurements

After examination by the ED physician, the patient and the ED physician completed separate paper-based questionnaires, with evaluation ratings of the tool on modified Likert scales. Nurses completed the same questionnaire as physicians when possible; however, it was recognized at the time of trial design that there would not always be a nurse–patient interaction after triage in which the handover report is relevant. All questionnaires were designed on the premise of optimizing completion (and therefore full participation) through highly simplified design. A validated usability tool design, for example, the System Usability Scale [22], was not used as the System Usability Scale addresses usability only, whereas it was important for the questionnaire in this study to address not only usability, but also usefulness of the tool. The Mobile App Rating Scale [23] is a validated tool for addressing usability and usefulness, but it would not have been feasible for HCPs to complete this multidimensional instrument in real time without disruption to their clinical routine; nor would it have been feasible for all the HCPs to complete a training exercise in the Mobile App Rating Scale before its use, as recommended in the study by Stoyanov et al [23]. Considering these factors, there is currently no published validated instrument for addressing usability and usefulness for a tool specifically designed for increasing patient–HCP rapport around self-reported medical history and symptom taking. The primary hypothesis, that the current tool had the potential to facilitate bidirectional patient-clinician conversation and rapport building, was assessed through the patient questionnaire items (originally in German): (1*)* “Was the digital history-taking experience engaging?” (2) “Could you understand the questions asked by the tool?” (3) “Do you think that the tool could facilitate better treatment at the ED?” and (4) “Did you feel better understood when speaking to the physician because they were already aware of your medical problem?” Corresponding questions for nurses and physicians assessed the HCP perspectives of rapport, as described in the *Results* section. The secondary hypothesis, that the tool would have the potential to facilitate documentation and thereby save HCP time, was assessed through the following HCP questionnaire items: (1) “Would the tool provide medically helpful information?” and (2) “Would the tool (as currently implemented) save time?”

Qualitative insights were also collected in the study, with a focus on usability and user interface improvements. To collect data on physicians’ interaction with the HCP handover, we applied methods of contextual inquiry [24]: observations, contextual interviews, and cognitive walkthroughs. This combination of methods was chosen for the primary purpose of enabling iterative co-design and development of products with users. The action-oriented study design allowed the rapid implementation of improvements based on phase 1 insights, resulting in a context-optimized patient tool and HCP interface in phase 2. The qualitative insights were collected by a multidisciplinary team consisting of a user experience researcher (AS), an interface designer in the product development team (JN), and the clinician research physician (JSB). Qualitative data were collected using multiple modalities (observational data, contextual interviews, and cognitive walkthroughs) to triangulate insights and reduce bias caused by any rapport built between participants and researchers throughout the study. Qualitative data were collected on a convenience sampling basis in 3 days of contextual interviews and cognitive walkthroughs at the start of phase 1 (conducted by AS, JN, and JSB) and at the start of phase 2 (conducted by JSB). Multimedia Appendix 1 contains the *User Research Guide*, which provides a detailed description of the qualitative methods applied.

### Study Setting

The study was conducted in the ED of the Katharinenhospital, Klinikum Stuttgart, which is an adult tertiary referral and major trauma hospital in southwestern Germany. It provides interdisciplinary emergency treatment for between 100 and 120 patients per day. The center adopts the First View Concept [25], in which an emergency registrar or consultant sees each patient in an interdisciplinary approach. The center has 23 treatment rooms with central monitoring, a resuscitation room, a wound room, and a plaster room. It uses the internationally recognized ESI triage system to guide the treatment of ED patients according to medical urgency [21].

### Sample Size Determination

This study was designed for real-world tool optimization (in phase 1), followed by a preliminary observation assessment of the tool’s potential in the ED. It was also designed as a guide to a later larger trial and in line with literature on pilot study design [26,27]. Therefore, the sample size was estimated on the basis of having a sufficient number of patients to assess survey completion rate and to determine if there were any safety-related considerations that might be needed in a randomized controlled trial (RCT). The aspects that were piloted were as follows: (1) trialing of new procedures and enabling power calculations intended to be used in a later single- or multicenter RCT; (2) establishing how many patients and HCPs can be recruited and the feasible level of completed patient, physician, and nurse questionnaires; and (3) evaluating the general technical and logistical feasibility of a full-scale study, including questionnaire design and other data collection related issues.

### Data Analysis

The quantitative data were analyzed using standard Python (version 3.7.4; Python Software Foundation) statistical modules (SciPy module version 1.3.0) using descriptive statistics and the Mann-Whitney U test, a nonparametric test of statistical significance suitable for categorical data [28]. For statistical significance testing, the value of a (here a=0.05) was adjusted using the Bonferroni correction, a/*m*, where *m* is the number of questions evaluated for each group. For patients, *m*=6 and a*_corrected_*=0.0083; for HCPs, *m*=4 and a*_corrected_*=0.0125; and for the degree of patient self-sufficiency, *m*=1 and a*_corrected_*=0.05. The pairwise deletion method was used for handling missing data [29].

The qualitative data were analyzed using affinity diagrams, a clustering technique used in thematic analysis [30]. The categories of the affinity diagram were predefined as key areas of interest, outlined in the interview guide: usability, usefulness, comprehension, impact on patient-physician communication, clinical relevance of information, and fit with clinical workflows.

## Results

### Recruitment

**Figure 3:**
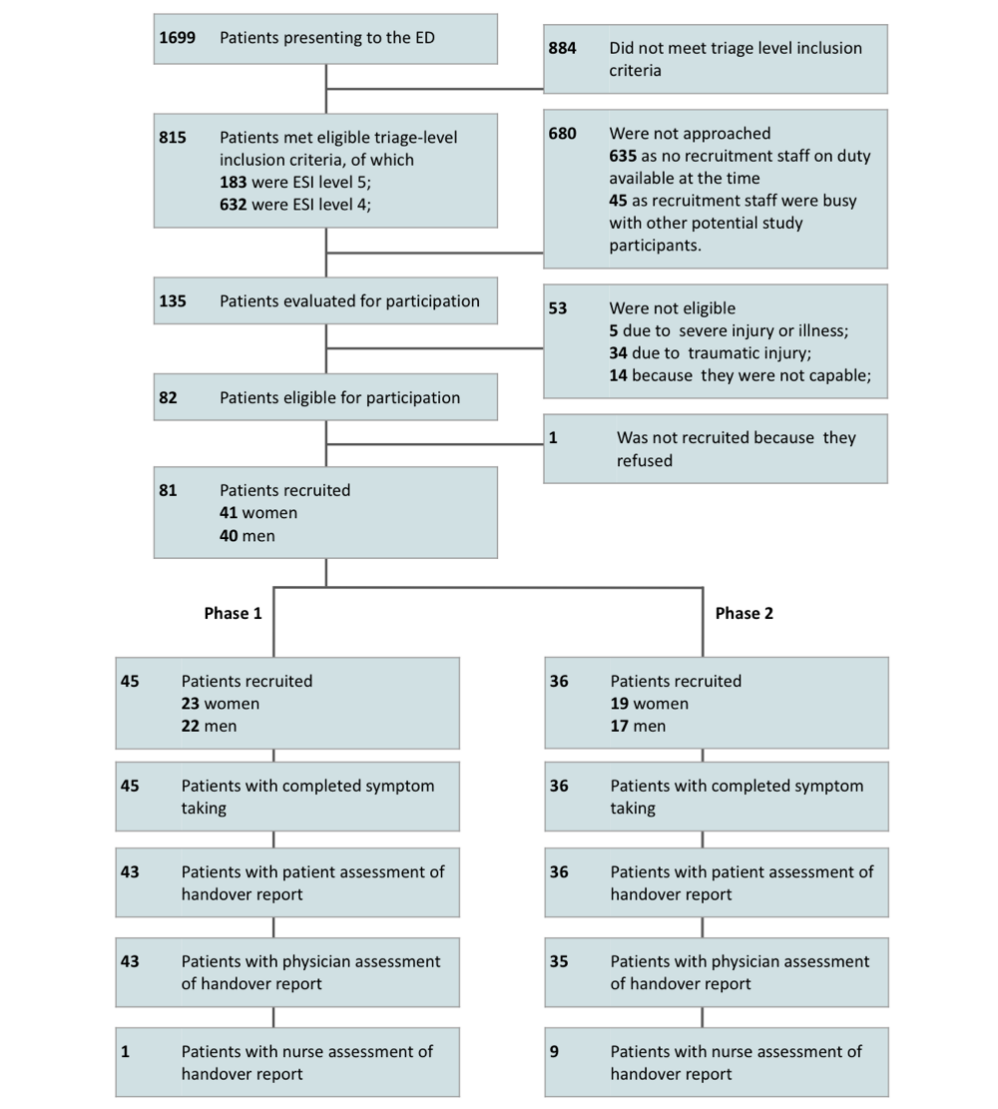
Participant recruitment flowchart. ED: emergency department; ESI: Emergency Severity Index.

The total number of recruited patients was 81 (41, 51%, women and 40, 49%, men), with 45 (56%) patients in phase 1 and 36 (44%) in phase 2 (Figure 3). Of the 81 recruited patients, there were 3 (4%) classified as ESI level 3, 77 (95%) classified as ESI level 4, and 1 (1%) classified as ESI level 5. A detailed description of the patient population is provided in Table 1, and the full results for each patient are presented in Table S1 of Multimedia Appendix 2.

**Table 1:** Description of the data completeness and medical subdiscipline of the final main diagnosis stratified according to study phase (N=81).

Group by study phase			All patients (N=81), n (%)		Patients included in phase 1 (n=45), n (%)		Patients included in phase 2 (n=36), n (%)
Age (years), mean (SD)			38.7 (15)		36 (15.9)		42.1 (13.1)
Completed patient evaluation questionnaire			81 (100)		45 (100)		36 (100)
Completed physician evaluation questionnaire			78 (96)		43 (96)		35 (97)
Completed optional nurse evaluation questionnaire			10 (12)		1 (2)		9 (25)
**Medical specialty classification (on the basis of** **emergency department** **discharge diagnosis)**							
	No diagnosis assigned	1 (1)		1 (2)		0 (0)	
	Orthopedics	12 (15)		9 (20)		3 (8)	
	Dermatology	5 (6)		5 (11)		0 (0)	
	Internal medicine, including specialties	28 (35)		13 (11)		15 (42)	
	Internal medicine: cardiovascular disease	4 (5)		1 (2)		3 (8)	
	Internal medicine: gastroenterology	4 (5)		1 (2)		3 (8)	
	Internal medicine: nephrology	1 (1)		1 (2)		0 (0)	
	Internal medicine: oncology	1 (1)		0 (0)		1 (3)	
	Internal medicine: rheumatology	1 (1)		1 (2)		0 (0)	
	Internal medicine, with no subspecialty	17 (21)		9 (20)		8 (22)	
	Neurology	20 (25)		8 (18)		12 (33)	
	Obstetrics and gynecology	1 (1)		1 (2)		0 (0)	
	Psychiatry	1 (1)		1 (2)		0 (0)	
	Surgery	10 (12)		6 (13)		4 (11)	
	Ear, nose, and throat	3 (4)		1 (2)		2 (6)	

### Baseline Data

Patient populations in the 2 study phases were similar (Figure 3 and Table 1): the mean age was 38.7 (SD 15.0) years for all patients, 36.0 (SD 15.9) years for patients included in phase 1, and 42.1 (SD 13.1) years for patients included in phase 2. The dominant medical classifications (by ED discharge diagnosis) were orthopedics, internal medicine, neurology, and surgery.

### Data Exclusion and Missing Data

For the 81 patients enrolled in the study, questionnaires were completed by 100% (81/81) of the patients (of whom 73/81, 90%, completed all questions), by 96% (78/81) of the physicians (78 completed questionnaires by the physicians for the 81 enrolled patients) and 12% (10/81) of the nurses (10 completed questionnaires by the nurses for the 81 enrolled patients; Figure 3 and Tables 1 and 2). Nurse questionnaires were only completed when they took part in symptom and history taking and when ED workload allowed. For all survey questions, the analysis approach was to report all data with respect to the number of responses to that survey question (ie, to use the pairwise deletion method for handling missing data [29], with the denominator in analyses being lower where there were missing data).

**Table 2:** Summary of patient, physician, and nurse ratings of the tool for phase 1, phase 2, and the phases combined. Two modified Likert scales were used: a 4-level Likert scale (1=Strongly disagree, 2=Disagree, 3=Agree, and 4=Strongly agree) and a 10-level Likert scale (1=Unlikely to 10=Highly likely). The mean and percentage positive ratings were calculated on the basis of the provided answers for each question. See Table S1 in Multimedia Appendix 2 for detailed data.

Statistic				All patients (N=81)		Patients included in phase 1 (n=45)		Patients included in phase 2 (n=36)
**Patient-provided ratings**								
	**1. Was the digital history-taking experience engaging?**							
		Patients, n (%)	81 (100)		45 (100)		36 (100)	
		Mean (SD), out of 4	3.4 (0.7)		3.2 (0.7)^a^		3.7 (0.5)^a^	
		Positive rating proportion^b^, n (%)	73 (90)		38 (84^a^)		35 (97^a^)	
	**2. Could you understand the questions asked by the tool?**							
		Patients, n (%)	81 (100)		45 (100)		36 (100)	
		Mean (SD), out of 4	3.4 (0.8)		3.2 (0.9)		3.6 (0.6)	
		Positive rating proportion^b^, n (%)	70 (86)		35 (78)		35 (97)	
	**3. Do you think that the tool could facilitate better treatment in the ED?**							
		Patients, n (%)	75 (93)		42 (93)		33 (92)	
		Mean (SD), out of 4	2.9 (1.0)		2.7 (0.9)		3.1 (1.0)	
		Positive rating proportion^b^, n (%)	51^c^ (68)		27^d^ (64)		24^e^ (73)	
	**4. Did you feel better understood when speaking to the physician because they were already aware of your medical problem?**							
		Patients, n (%)	73 (90)		40 (89)		33 (92)	
		Mean (SD), out of 4	3.0 (0.9)		2.9 (0.9)		3.1 (1.0)	
		Positive rating proportion^b^, n (%)	55^f^ (75)		28^g^ (70)		27^e^ (82)	
	**5. How do you rate the user experience provided to you in the tool (ie, its usability)?**							
		Patients, n (%)	79 (98)		43 (96)		36 (100)	
		Mean (SD), out of 10	7.3 (2.3)		7.4 (2.2)		7.2 (2.5)	
		Positive rating proportion^b^, n (%)	66^h^ (84)		36^i^ (84)		30 (83)	
	**6. Would you recommend the tool to others?**							
		Patients, n (%)	77 (95)		41 (91)		36 (100)	
		Mean (SD), out of 10	7.3 (2.3)		7.1 (2.3)		7.5 (2.4)	
		Positive rating proportion^b^, n (%)	60^j^ (78)		31^k^ (76)		29 (81)	
**Physician-provided ratings**								
	**1. Would the tool facilitate rapport with the patient?**							
		Patients, n (%)	78 (96)		43 (96)		35 (97)	
		Mean (SD), out of 3	2.8 (0.8)		2.8 (0.9)		2.9 (0.7)	
		Positive rating proportion^b^, n (%)	57^l^ (73)		29^i^ (67)		28^m^ (80)	
	**2. Would the tool provide medically helpful information?**							
		Patients, n (%)	78 (96)		43 (96)		35 (97)	
		Mean (SD), out of 4	2.6 (0.9)		2.6 (0.9)		2.7 (0.8)	
		Positive rating proportion^b^, n (%)	43^l^ (55)		21^i^ (49)		22^m^ (63)	
	**3. Would the tool (as currently implemented) save time?**							
		Patients, n (%)	78 (96)		43 (96)		35 (97)	
		Mean (SD), out of 4	2.2 (0.9)		2.3 (1.0)		2.2 (0.8)	
		Positive rating proportion^b^, n (%)	27^l^ (35)		16^i^ (37)		11^m^ (31)	
	**4. Would you recommend the tool to colleagues?**							
		Patients, n (%)	77 (95)		43 (96)		34 (94)	
		Mean (SD), out of 10	5.7 (1.9)		5.4 (2.2)		6.1 (1.4)	
		Positive rating proportion^b^, n (%)	41^j^ (53)		20^i^ (47)		21^n^ (62)	
**Nurse-provided ratings**								
	**1. Would the tool facilitate rapport with the patient?**							
		Patients, n (%)	10 (12)		1 (2)		9 (25)	
		Mean (SD), out of 4	3.7 (0.5)		4.0^o^		3.7 (0.5)	
		Positive rating proportion^b^, n (%)	10^p^ (100)		1^q^ (100)		9^r^ (100)	
	**2. Would the tool provide medically helpful information?**							
		Patients, n (%)	10 (12)		1 (2)		9 (25)	
		Mean (SD), out of 4	3.4 (0.5)		3.0^o^		3.4 (0.5)	
		Positive rating proportion^b^, n (%)	10^o^ (100)		1^p^ (100)		9^q^ (100)	
	**3. Would the tool (as currently implemented) save time?**							
		Patients, n (%)	10 (12)		1 (2)		9 (25)	
		Mean (SD), out of 4	3.2 (0.8)		3.0^o^		3.2 (0.8)	
		Positive rating proportion^b^, n (%)	8^p^ (80)		1^q^ (100)		7^r^ (78)	
	**4. Would you recommend the tool to colleagues?**							
		Patients, n (%)	10 (12)		1 (2)		9 (25)	
		Mean (SD), out of 8	7.8 (0.8)		8.0^o^		7.8 (0.8)	
		Positive rating proportion^b^, n (%)	10^p^ (100)		1^q^ (100)		9^r^ (100)	
**Patient self-sufficiency**								
	**Degree of patient self-sufficiency (on the 4-level scale of assistance: 1=high, 2=medium, 3=low, and 4=none).**							
		Patients, n (%)	80 (99)		44 (98)		36 (100)	
		Mean (SD), out of 4	3.1 (1.0)		2.8 (1.1)		3.4 (0.7)	
		Proportion who received little or no help, n (%)	61^s^ (76)		28^t^ (64)		33 (92)	

^a^Statistically significant difference in Likert scores, according to the Mann–Whitney U test.

^b^Percentage of positive ratings on the 4-level Likert scale, that is, the percentage of *3* and *4* ratings.

^c^n=75.

^d^n=42.

^e^n=33.

^f^n=73.

^g^n=40.

^h^n=79.

^i^n=43.

^j^n=77.

^k^n=41.

^l^n=78.

^m^n=35.

^n^n=34.

^o^SD not defined as the group size is 1.

^p^n=10.

^q^n=1.

^r^n=9.

^s^n=80.

^t^n=44.

### Qualitative Learnings

The qualitative learnings are summarized in Table 3.

**Table 3:** Summary of qualitative insights.

Study phase, theme, and users			Observation
**Phase 1**			
	**Usability**		
		Patients	Operating the digital tool and finishing the question flow was successful for most patients
	**Usefulness**		
		Physicians	Provision of additional patient information (past medical history, medications, and allergies) was considered very important to the overall utility of the tool
		Physicians	Treating physicians expected the tool to collect patient information beyond medical history and acute complaints, for example, “collecting a social anamnesis for a full picture of patient background”
		Physicians	The ability to read a patient’s history and symptoms before consultation was generally described as making the interaction with the patient more pleasant for both patient and physician (rapport) and making treating physicians more prepared
		Physicians	The tool primarily provided clinical value for newly occurring problems. It provided less added value for the following: patients with severe or visible trauma patients with complaints resulting from chronic conditions patients with multiple comorbidities
		Physicians	The highly mixed patient population (age, language, digital literacy, medical complaints, and socioeconomic status) makes the emergency department a challenging setting for the tool
		Physicians and nurses	Greater integration of the tool with the electronic health record systems and clinical workflows is desirable
	**Comprehension**		
		Patients	The language used in the tool was difficult for several observed users to understand because of the following: limited German language ability (nonnative German speakers) limited language reading level (some native German speakers)
		Patients	Several observed users had a low ability to articulate complaints in a manner that the tool could process (when using either medical or layperson’s terms). This related to the following: difficulty in localizing pain to provide the tool precise answers to localization questions difficulty entering multiple symptoms separately general difficulty in verbalizing symptoms difficulty finding accurate synonyms in the tool for the feelings they were experiencing
**Phase 2 (only new or changed insights recorded)**			
	**Usefulness**		
		Physicians and nurses	The V2 tool asked patients to provide information on past medical history, medications, and allergies, thereby providing a fuller picture of the patient’s health beyond their acute symptoms and was recognized by physicians and nurses as more beneficial and better supporting the physician–patient consultation
		Nurses	Nurses were enthusiastic regarding time-saving potential in history taking with V2 tool features

### Summary of Changes Made Between the V1 and V2 Systems

We made three changes to the V1 system to create the V2 system based on qualitative observations and quantitative findings:

1. The addition of 3 free-text input fields, where the patient can supply initial information on their medical history, current medications, and allergies: This change was requested by ED physicians so that patients would have the opportunity to pass on information in their own words that was not always collected by the tool’s question flow. This was implemented in a manner that did not change the core AI-based symptom assessment. This change affected the information entered into the patient-facing tool and the information presented on the HCP-facing tool.
2. Improvement of the user interface at the transition between patient information and symptom assessment: A minor rephrasing was made to the patient-facing tool in response to a small number of patients who reported that they misunderstood a specific direction of how to proceed from one step to the next in the early phase of the assessment (a clear direction originally developed in an earlier English language prototype had been poorly expressed in German).
3. Fixing a minor bug affecting the HCP handover report: A minor bug was removed that had resulted in a small number of handover reports not accurately displaying the transcript of questions that Ada asked the patient alongside the patient response.

The changes were anticipated to have a minor impact on the evaluation of the tool. The patients’ ratings of the tool (Table 2) improved by 9.5% and those of the physicians by 9.1% across all ratings with a significant improvement (*P*=.003; a*_corrected_*=0.0083) in the responses to the patient question “Was the digital history-taking experience engaging?” The changes in nurse ratings of the system between phase 1 and phase 2 are reported in Table 2 and Table S1 of Multimedia Appendix 2; however, there were insufficient nurse assessments in phase 1 to allow statistical significance testing.

### Patient, Physician, and Nurse Ratings of the Tool

Patients were positive or highly positive (73/81, 90%) about how engaging the tool was to use, its comprehension (70/81, 86%), its usability (68/81, 84%), its ability to facilitate understanding and rapport with the HCPs (61/81, 75%), and about recommending the tool to peers (68/81, 84%; Table 2 and Table S1 of Multimedia Appendix 2). Likewise, 73% (57/78) of the physician ratings were positive or highly positive about the tool’s potential for facilitating understanding and rapport with patients.

Nurses were even more positive or highly positive about the tool’s potential for facilitating understanding and rapport with patients (10/10, 100% of nurse ratings), about the helpfulness of the medical information provided (10/10, 100% of nurse ratings), about the tool’s time-saving potential (8/10, 80% of nurse ratings), and about recommending the tool to peers (10/10, 100% of nurse ratings).

### Subanalyses by Medical Specialty

A post hoc subanalysis by medical specialty of ED discharge diagnosis is shown in Table S1 of Multimedia Appendix 2 and described in detail in Multimedia Appendix 2. This subanalysis showed that there was lower comprehension of the tool by patients receiving care from a neurologist and that these patients also gave lower ratings to the conversation facilitation provided by the tool. Physicians rated the level of helpful information, the time-saving utility of the report, and the likelihood of them recommending the system more positively for internal medicine handovers than for handovers for all recruited patients or for other specialties.

### Degree of Patient Assistance Provided

Across both phases, most patients were able to use the tool with little or no assistance (62/81, 76%). This measure improved significantly between phase 1 (52/81, 64%) and phase 2 (74/81, 92%; *P*=.003; a*_corrected_*=0.05).

### Variability of Physicians’ Perceptions

Patient symptom and history data handover were evaluated for 96% (78/81) of the patients by 24 different ED physicians. Qualitative interviews with physicians in phase 1 of the study revealed a number of physicians who were exceptionally enthusiastic about the performance of the system, ie, they exhibited an *early adopter* mentality. The quantitative analysis of the distribution of Likert scores (Figure S1 in Multimedia Appendix 2) supported these qualitative findings, in that there is a skewed distribution of physician scores, with 2 physicians (one of whom evaluated handover reports for 6 patients) providing highly positive evaluations of the tool.

### Sample Representativeness

The mean age of patients was 38.7 (SD 15.0) years, which is comparable to the mean age of 41.8 (SD 19.3) years reported in a recent cross-sectional study of patients attending German EDs [31]. The study showed that those who refused to participate in the general ED study, which did not involve assessment of a digital health tool, were older (difference in mean ages 3.6 years) than those who agreed to participate. Our study had a moderately lower participant age group than that reported in the study by Scherer et al [31] (difference in mean ages 3.1 years) and this might reflect, to a minor degree, older patients being less willing to try a digital tool. However, 24% (19/81), 7% (6/81), and 4% (3/81) of the patients in our study were aged >50 years, >60 years, and >70 years, respectively. Overall, the patient population closely reflects the proportion of the German ED patient population that agrees to participate in studies and is reflective of the overall self-referred walk-in German ED patient population [31,32].

### Larger Study Planning

A 100% (81/81) response rate was achieved for the patient questionnaire completion and a very high response rate also for the physician questionnaire (78/81, 96%). This was made possible through diligent tracking and follow-up of the patients and physicians; a planned and systematic approach was taken because high questionnaire completion was seen as a criterion for judgment of the feasibility of an RCT, after tool optimization, for regulatory approval. The nurse questionnaire completion rate was much lower (10/81, 12%); it had been understood in planning that (1) not every patient has an interaction with nurses that would be meaningful to assess, (2) the complexity and variability of the timings of the nurse interactions with the patient provided no single time point at which the nurse questionnaire could be completed, and (3) the nurses’ workload pressures would mean that completion would be an additional burden. For these reasons, completion was optional for the nurses in the study design. We accept that firm conclusions on nurse perception cannot be made on the basis of completed nurse questionnaire for 12% (10/81) of the patients; however, we report these data for completeness.

## Discussion

### Principal Findings

The performance of the system was evaluated with respect to two hypotheses: the primary hypothesis was that the tool had the potential to facilitate bidirectional patient-clinician conversation and rapport building and the secondary hypothesis was that the tool would have the potential to facilitate documentation and thereby save HCP time.

There was a strongly positive rating by patients, physicians, and nurses of the tool as an aid in patient–clinician conversation, communication, and rapport building. The proportion of physicians who were positive or highly positive about recommending the system to their peers (in 41/77, 53%, of physician ratings) was not as high as it was for patients and nurses (63/81, 78%, of patient ratings and 10/10, 100% of nurse ratings). Overall, physicians had mixed views on the degree of helpful clinical information and time-saving potential of the clinical handover report. Underlying the mixed results in the physician ratings (Table S1 of Multimedia Appendix 2) were a large number of patients for whom the ED physicians described the presence of a *visual diagnosis*, that is, one that was immediately apparent by simply looking at a patient and for whom patient-directed symptom taking, in whatever form it is designed, is unlikely to save history-taking and recording time. For some other patients, the tool did not adequately draw together and summarize a highly complex, sometimes multimorbid medical presentation in a manner that made it useful for physicians. Both qualitative findings and quantitative analysis revealed a subset of highly enthusiastic early-adopter physicians, as has been recognized in other studies [33], who were highly positive about the history and symptom-taking information provided by the tool and for its potential to improve rapport, to provide helpful clinical information, and to save ED time.

The relatively lower comprehension of the tool by patients receiving care from a neurologist and the lower rating of its conversation facilitation may reflect effects of neurological symptoms on their use of the tool. This could be related to inherent challenges of achieving an acceptable user interface for these patients for self-history and symptom recording, or it may be that the tool requires further specific optimization for this patient group—further quantitative and qualitative study of this group in a larger study is required before definitive conclusions can be drawn. Our interpretation is that the positive or highly positive evaluations from physicians for internal medicine handovers is likely due to the system’s AI reasoning engine being better at directing the question flow for conditions that have many subtle interlinked clinical symptoms.

An aim for the development of a waiting room patient history–taking and HCP handover tool is that it should be easy to use without assistance. Many patients visit only once; therefore, they would have little opportunity to learn to use the tool over time. In this study, a degree of patient assistance was provided (where needed) by the recruiting clinical research physician (JSB), and in all cases the degree of assistance provided was documented. Most (62/81, 76%) of the patients required little or no help with the use of the tool, and in some subspeciality, patients were highly independent using the tool (76/81, 94%, in internal medicine with no subspeciality), whereas 67% (54/81) of patients receiving care from an orthopedist required little or no help. A priority in the future development of the prototype will be to adapt the reasoning engine and the user interface to minimize the level of help required for all patient groups, and this may involve making the tool available in the patient’s primary language. It is known that the degree of patient eHealth literacy can be an enabler or a barrier for patients, especially for older adults [34-36]. Internationally, particularly in low- and middle-income countries, eHealth literacy of HCPs, including medical and paramedical staff, may be a barrier to widespread adoption [37,38]. We propose that the potential limitation to adoption as a result of low eHealth literacy, for both patients and HCPs, is best resolved through user-focused design and development, followed by user-focused mixed methods pilot evaluation (as in this study). After tool refinement, multicenter RCTs and real-world–performance modeling are appropriate and necessary.

The study incorporated 2 study phases, providing an opportunity to rapidly modify a prototype tool’s design based on feedback from patient and physician evaluations and to further evaluate the modified tool. A comparison of tool ratings showed statistically significant improvements in performance between the V1 and V2 tools in two evaluation categories: (1) *how engaging or interesting patients found the tool to use* and (2) *the degree of self-sufficiency of the patients in using the tool*. These changed scores reflect improvements made in tool usability and functionality. Many useful insights on tool performance and usability were obtained in the study. Only a relatively small number of optimizations could be executed between the V1 and V2 tools because of the need to prioritize only those in-study changes that could be completed within the possible study recruitment period. The remaining insights, including those regarding the V2 tool, will be used in later prototype development and optimization. The qualitative findings broadly reflect the quantitative findings of the study, particularly with respect to the groups of patients who benefit most from the tool. Importantly, the qualitative findings also provided a means in the study to iterate on the tool design and to gain deeper insights into further improvements that can be made in tool design, particularly with the interface and language level.

Open questions for future research include the potential for handover of home-completed digital symptom and history taking to HCPs for rapport and communication building and time saving and the potential of the tool in a general nonemergency setting. Other open questions relate to the precise contribution that the evaluated tool or technologies related to it could make at times of substantial impact of COVID-19 on health care provision. This study was conducted in a period of low COVID-19 case numbers. It was recognized early in the COVID-19 pandemic that eHealth tools were needed to reduce the likelihood of health care facilities being overwhelmed and to assist in providing health care without face-to-face contact [39,40]. Although eHealth systems contributed substantially throughout the pandemic, they did not provide a panacea for care delivery [41].

### Limitations

This was a single-site study in Germany, and the findings may not be generalizable to other facilities or to other countries. The patient and physician quantitative evaluations were not influenced by selection bias; however, because the nurse evaluations were completed on a voluntary basis, the influence of selection bias cannot be excluded (9/10, 90% of the nurse evaluations related to study phase 2). The study questionnaire was not based on a previously validated instrument; therefore, response bias cannot be excluded. The pairwise deletion approach was used for handling missing data [29], which results in the analysis being based on different sets of data with different sample sizes. The approach of modeling the missing data and estimating missing values is an alternative and is considered the optimal approach by Kang et al [29]. The qualitative methods were applied to a smaller subgroup of users (patients and HCPs) in the first days after tool introduction and cannot be taken to be fully representative of the full study population. This study explored tool use in the German language and in the German setting only, and the sample size was relatively small, given the resource constraints within the ED. The study did not include patients receiving care from a pediatrician. Infection control with patients using a tablet computer in the ED could be challenging for large-scale deployment. This study explored the early phase after prototype system implementation, training, and first experience of its application by users. Although minimally relevant for ED patients, the skill and speed of ED physician and nurse use of the new tool and their perception of its performance are likely to change over time. Nurse questionnaires were completed for 12% (10/81) of the patients; therefore, firm conclusions cannot be drawn about their perceptions of the tool. Despite the limitations, our approach allowed us to investigate implementation at this single site in considerable depth.

### Comparison With Prior Work

There are no previous studies describing similar Bayesian digital history-taking and handover systems in the literature. Arora et al [42] explored patient impressions and satisfaction of a self-administered, automated medical history–taking device in the ED and also reported high levels of patient enthusiasm and potential for building rapport. However, handover to HCPs was not carried out in the study; nor did the tool have a system to enable handover. A protocol has been published for a prospective cohort study of a self-reported computerized medical history–taking tool (Clinical Expert Operating System [CLEOS]) [43]. The CLEOS tool has also been evaluated in an observational study that looked at history taking using the tool subsequent to clinician history taking [44,45]. The focus of these studies was on history taking for the sake of record completeness and less so on the potential for building rapport. The CLEOS tool is fundamentally different in its interface with patients and in the underlying logic that drives patient questioning. The CLEOS tool uses a decision tree approach, whereas the tool evaluated in this study (from Ada) uses a Bayesian network that is defined upon a medical knowledge base and on which approximate inference is carried out, followed by information theoretical methods used to decide which questions to ask the user. There is no simple answer as to whether decision trees or Bayesian network approaches are superior for patient symptom and history taking. One argument in support of the Ada approach is that it aims to ask specific questions based on a large array of possible conditions that a patient may have to gather a highly personalized medical history. This would require an unmanageable size of decision trees, leading to inconsistencies, imbalances, and low accuracy [46].

### Implications for Clinicians and Policy Makers and Implications for Future Research

It is recognized that empathy and rapport can be lacking in EDs, both of which are important for staff stress levels and satisfaction and for patient outcomes and empowerment [1,6,7,47,48]. Although a digital tool can never be a complete solution to improve human-to-human empathy, this study has nonetheless demonstrated patient and HCP enthusiasm for the rapport building and conversation facilitation enabled through such tools. This study only evaluated a prototype, and further developments are required to facilitate patient–HCP conversations and optimize rapport in a manner that is complete (ie, clinically helpful information for every patient), streamlined, and seamless (ie, no additional tasks or systems for clinicians). This mixed methods pilot study adopted a user-driven approach, and the results show that the tool has large potential for rapport and communication building. This study has provided a foundation for the further development of the tool, which will be followed by an RCT of a completed tool.

Although the results of this study were definitive regarding the potential for rapport and conversation facilitation in the ED, they were equivocal on the potential for the tool, as it is currently implemented, to save clinician time. Clinicians could only identify opportunities for saving time in selected types of patient presentations, namely internal medicine and surgery. Nurses were more positive about the general potential of the tool for clinician time saving. It is recognized that further development of the prototype is required if the aim of the completed approved regulatory product is to deliver definitive physician time savings for all patients. The role of a patient history and symptom-taking tool is not only to save clinician time, but to also contribute to documentation accuracy and completeness. It is known that medical performance reduces with stress and overstretching in the ED and is likely to result in more errors, including in documentation [8,31]. It was not possible to measure the contribution of the tool to documentation completeness and accuracy in this study.

### Conclusions

The patient- and HCP-reported data from this mixed methods pilot study supported the primary hypothesis that the tool improved rapport between patient and clinician and improved patient-clinician communication. Mixed methods trials are powerful approaches to gain insights into tool potential and to optimize a tool for a particular clinical setting. However, confirmatory evaluation is needed in RCTs and in different ED settings. Notably, patients felt better understood and the tool had utility in efficiently recording their symptoms. Nurses perceived the tool as having the potential to save time through workflow efficiency. Some physicians were enthusiastic about the potential to improve patient interaction and about the tool’s benefits in symptom and history taking. Results regarding the secondary hypothesis of documentation assistance and time saving in the ED were more equivocal, but there was potential for time saving in some medical subspecialties, for example, internal medicine and surgery. Insights from this study will be used for further prototyping and research to extend the range of patients for whom the tool can provide support. The tool is based upon an existing and regularly maintained medical reasoning engine and therefore is a sustainable technological approach for history and symptom taking. The tool is readily adaptable to other related settings in which patient self-symptom and history taking and conversation support are relevant, including at home and at primary care and specialist clinics (eg, specialist rare diseases clinics).

## Appendix

Multimedia Appendix 1 User research guide.

Multimedia Appendix 2 Results supplement with a subanalyses by medical specialism and the Likert score distribution for the emergency department physician.

## Abbreviations

AI: artificial intelligence
CLEOS: Clinical Expert Operating System
CONSORT-AI: Consolidated Standards of Reporting Trials–Artificial Intelligence
ED: emergency department
ESI: Emergency Severity Index
HCP: health care provider
RCT: randomized controlled trial
SPIRIT-AI: Standard Protocol Items: Recommendations for Interventional Trials–Artificial Intelligence
