# Supplementary material for: Improving Emergency Department Patient-Physician Conversation Through an Artificial Intelligence Symptom-Taking Tool: Mixed Methods Pilot Observational Study
Source: JMIR Form Res. 2022 Feb 7;6(2):e28199. doi: 10.2196/28199 (PMC8861871; doi:10.2196/28199)
Supplement: Multimedia Appendix 1 [file formative_v6i2e28199_app1.pdf]

User research guide -

Mixed-methods pilot observational study

Context

The user research plan is supporting the mixed-methods pilot observational study and its objective to evaluate a digital history and handover system prototype, which includes a patient-facing tool and HCP-facing tool for symptom and history taking.

Phase 1 refers to the initial implementation of the patient- and HCP-facing tools (version V1). In this particular phase, the focus of the evaluation will be predominantly qualitative, using a range of different methods in order to collect insights for interface improvements.

Phase 2 refers to the second stage of the study in which, based on feedback from Phase 1, the modified system (version V2) was further evaluated. The evaluation will have a more quantitative focus looking at usability and usefulness in facilitating patient-HCP conversation and rapport formation in the ED setting.

Study Design

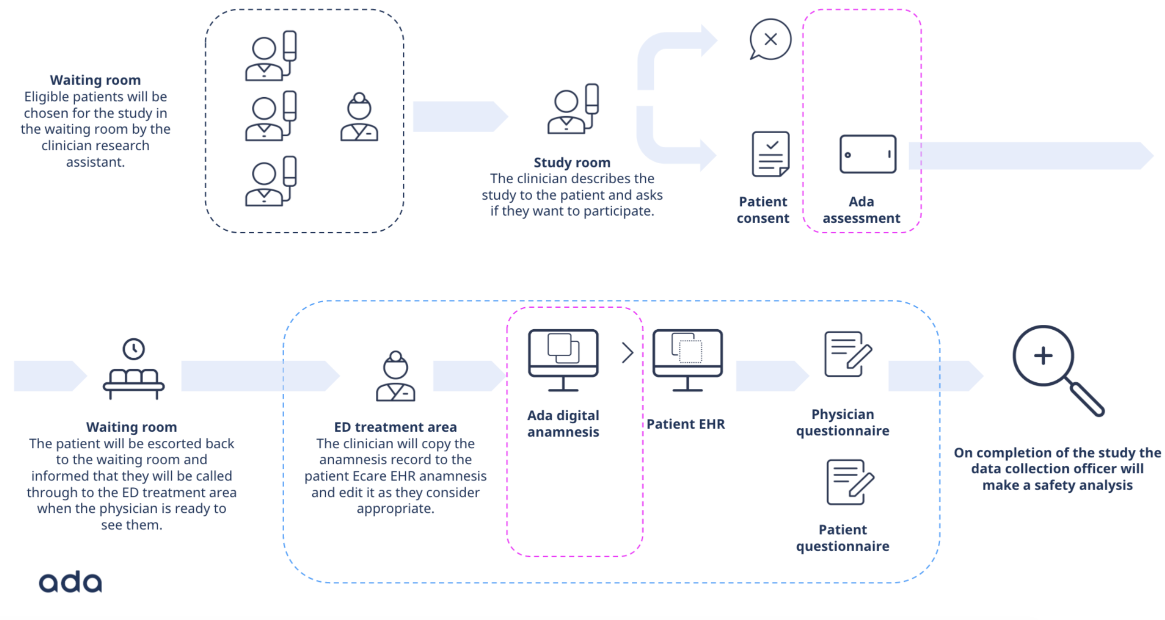

Overall Research Objective (Phase 1 & 2)

Main objective of the mixed-methods approach was to gather insights to inform interface improvements connected to the study objective to explore the potential of enhancing patient-HCP communication in an ED setting with a history and symptom taking tool.

In line with the main objective, three key areas have been identified to be considered in depth, which are the following:

Key areas:

1. Usability of the patient tool
2. Quality of patient input
3. Usefulness and relevance of the HCP-tool

Method Selection

A range of methods were considered and deemed appropriate to fit the mixed-methods approach of the pilot observational study. The methods were selected to fit the study objective as well as the particular context of the ED. The ED is a work environment which is dynamic and difficult to predict. The three methods allow the interdisciplinary team consisting of a user experience researcher, interface designer and clinician research physician to collect rich contextual information.

1. Shadowing
   Observations collected in the real-life context based on the What-How-Why method.

2. Contextual Interviews
   Interviews conducted with users using a product in a real use scenarios throughout early stages of product development.

3. Walkthroughs
   Conversations with users performing specific tasks in order to evaluate usability and usefulness of a particular product.

Participant Selection

The nature of an ED environment requires the participant selection to be based on opportunistic sampling.

- Shadowing takes place when ED staff and patients have consented to it
- Contextual interviews take place when the work situation in the ED allows for physicians and nurses to be interviewed.
- Walkthroughs are planned to take place when
  - Patients' health status allows and they have given their consent after having been approached by the clinician research physician.
  - Physicians' and nurses' workload in the ED allows

1. Shadowing

This short shadowing guide aims to guide any of the members of the interdisciplinary team to collect insights for the improvement of both tools.

It is important to mention that the key element of shadowing is that the person engaging in it acts as an observer only. There is no interaction with the research subject (i.e. HCP, nurse or patient), it is purely observational. For capturing observations, the What-How-Why method is recommended to gain a deeper level of understanding by noting findings from concrete observations to abstract interpretations.

Helpful aspects to note down when doing shadowing:
- **What** is observed
    - Who is involved
    - When is it taking place
- **How** is it done
- **Why** is it done

2. Contextual Interview guides (semi-structured)

This unstructured interview guide aims to guide in-person interviews with HCPs, physicians and nurses, using the HCP-facing tool. The questions have been selected as they aim to collect information about the key areas referred to in the section above as well as background information (needs, goals, motivations) about role-specific processes and workflows.

This is not an exhaustive list of questions, yet will help facilitate a conversation about existing processes and workflows in the ED setting as well as touch upon the relevance of the HCP-facing tool within those.

Physicians

HCP Processes/Workflow
- When is the ideal time to read through the patient report?
- What does a conversation with a patient look like?
- What information do you know about the patient upon their arrival?

- Where do you obtain this information from?
- What information and in what format do you have about the patient's case prior to the consultation?
- What tools do you use (both offline and online)?
- How long does it usually take you to do the consultation?
- What are you trying to uncover with the consultation?
- How do you document the results of consultation?
- What are the next steps for you and the patient?
- Are other colleagues involved at any point of time throughout the consultation?

HCP-facing/Patient-facing Tool
- Can you reflect upon your interaction with the HCP-facing/patient-facing tool?
- How relevant was the handover report for the patient case?

Opportunities for Tool Improvements
- Is the report useful in the way it is currently compiled?
    - If yes, what is valuable about it?
    - If not, why not?
- Is the information hierarchy relevant for/ supporting the current way of collecting anamnesis?
    - If yes, why?
    - If not, why not?

Information
- Is there any crucial information missing?
- Is there any information which is currently collected/displayed redundant in your opinion?
- How would you assess the quality of the information provided?

Documentation
- Is this documentation facilitating subsequent patient conversation?
- Is there anything which could improve the documentation?
- Is this documentation clinically relevant for colleagues?
- Does this documentation require additional editing?
    - If yes, why?
    - If not, why not?

Efficiency
- Does this tool support/ improve current workflows?
    - If yes, why?
    - If not, why not?

Communication

- Can the tool help facilitate patient-HCP communication?
- If yes, how and why?
- If not, why not?

EHR Workflow
- What is key to making this tool a success in your workflow?
- Where do you see the biggest challenges using the tool?
- Where do you see the value, if any?

Nurses

Processes/Workflow
- Can you describe the steps you take when admitting a patient/recording patient complaints?
- Do you perform a triage? Which scoring system do you use?
- What is the necessary information you collect about the patient?
- How do you present the patient's case to the doctor?
- What are the steps you take after the doctor is finished with consulting the patient?

Patients

Experience with Patient-facing Tool
- How was the experience using a digital history and symptom taking tool in general?
- Was there anything difficult about using the history and symptom taking tool?
- How easy was it to understand the questions?
- How easy was it to answer the questions?
- How easy was it to understand the language being used?
- Anything you would like to see improved?
- Anything you particularly liked about the tool?

3. Walkthroughs

This short guide for walkthroughs aims to facilitate conversations with all study subjects (i.e.

physicians, nurses and patients) when collecting valuable feedback about the HCP-facing and patient-facing tool's usability and usefulness.

Let participants perform a task or a series of tasks with either tool and keep the following questions in mind.

1. Will the participant be able to complete the task?
2. Is the right action clear to the participant?
3. Is it clear to the participant which action leads to which outcome?
4. Can the participant clearly see progress towards completing the task?
5. Are there any emotional reactions (frustration, doubt, confusion, accomplishment, ease etc.) by the participant while performing the task?
