# Supplementary material for: Improving Emergency Department Patient-Physician Conversation Through an Artificial Intelligence Symptom-Taking Tool: Mixed Methods Pilot Observational Study
Source: JMIR Form Res. 2022 Feb 7;6(2):e28199. doi: 10.2196/28199 (PMC8861871; doi:10.2196/28199)
Supplement: Multimedia Appendix 2 [file formative_v6i2e28199_app2.docx]

# Supplemental Material

## Results Supplement - Subanalyses by medical specialism

A subanalysis by medical specialism of ED discharge diagnosis was carried out for all medical specialisms for which there were 10% or more of the patients included (Internal Medicine, Internal Medicine (with no subspecialism), Neurology, Orthopaedics, and Surgery) is presented in (**Data Supplement Table 2**). Patient self-sufficiency in the use of the tool was relatively lower in Neurology (66.7% requiring little/no help) (compared to 76.3% for all patients).

In terms of patient evaluations, the subanalysis groups had similar ratings to the all-patient analysis, except in the following themes, were there was greater than 10% difference between the patients in the specialism and the full study population: (i) 75.0% of Neurology patients (compared to 86.4% of all patients) gave a positive/strongly positive evaluation for the understandability of tool questions; (ii) 100.0% of Orthopaedics patients (compared to 68.0% of all patients) gave a positive/strongly positive evaluation for their view of the potential for the tool to facilitate better treatment in the ED; (iii) 61.1% of Neurology patients (compared to 75.3% of all patients) gave a positive/strongly positive evaluation for the tools ability to increase their being understood when speaking to the physician; (iv) 100.0% of Surgery patients (compared to 83.5% of all patients) gave a positive/strongly positive evaluation for the tools usability; and, (v) 100.0% of Surgery patients (compared to 77.9% of all patients) would recommend the tool to fellow patients.

For physician evaluations, there were similar ratings in the subanalyses to the all-patient analysis, except in the following themes: (i) 66.7% of Internal Medicine (all) and 66.7% of Surgery patient handovers (compared to 55.1% of all handovers) gave a positive/strongly positive evaluation for the provision of medically helpful information; (ii) 50.0% of Internal Medicine (with no subspecialism) and 55.6% of Surgery patient handovers (compared to 34.6% of all handovers) gave a positive/strongly positive evaluation for the potential for the tool to save time for the physician; (iii) 70.4% of Internal Medicine (all), 68.8% of Internal Medicine (with no subspecialism) and 100.0% of Surgery patient handovers (compared to 53.2% of all handovers) would positively/strongly positively recommend the tool to other physicians.

**Supplemental Figure 1.** ED-physician average Likert score distribution


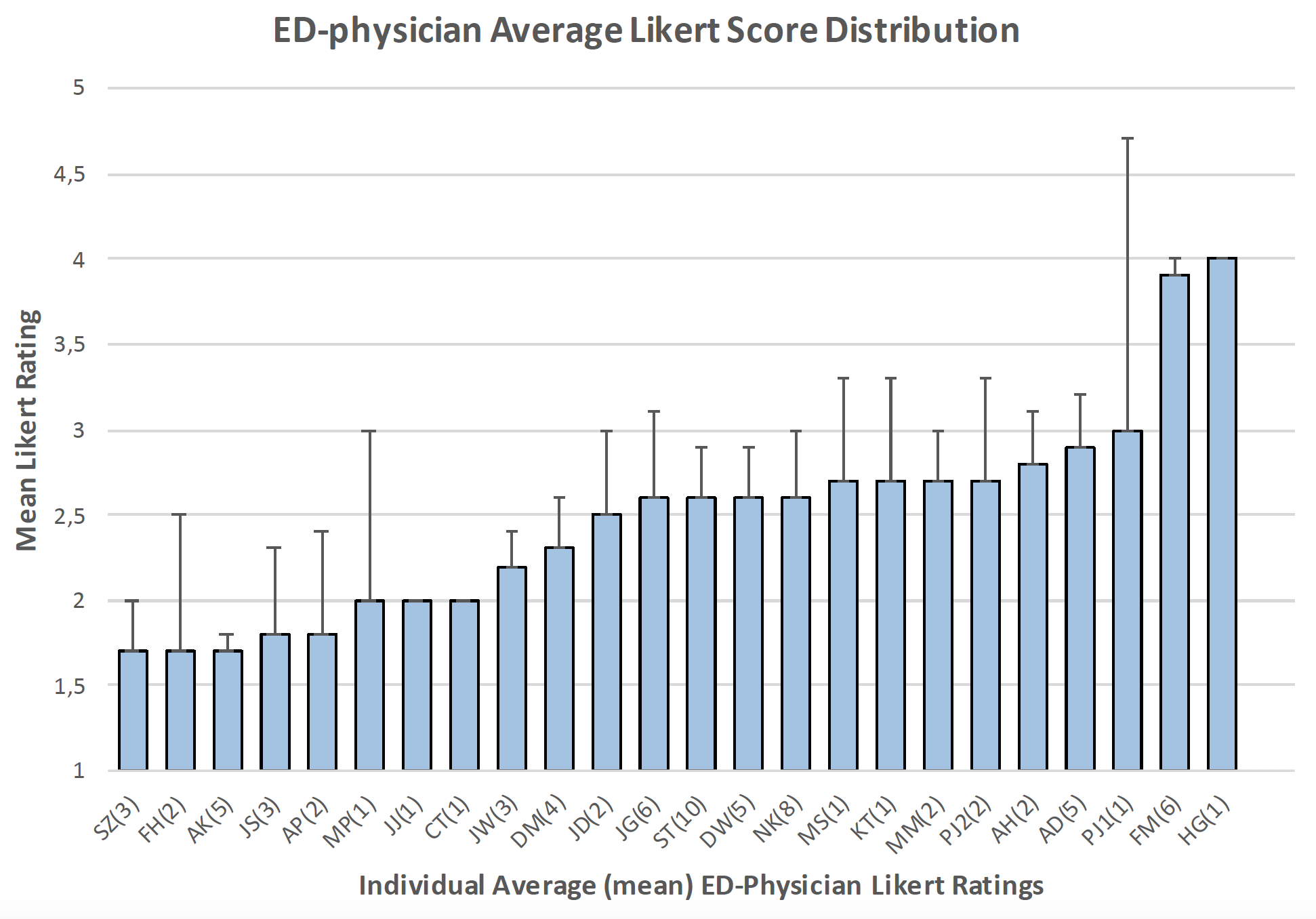


**Supplementary Table 1** The data for each patient and for each phase of the study.

| **ID** | **Study phase** | **Discharge diagnosis** | **Patient provided ratings** | | | | | | **Physician provided ratings** | | | | | | | | **Self-sufficiency** |
| --- | --- | --- | --- | --- | --- | --- | --- | --- | --- | --- | --- | --- | --- | --- | --- | --- | --- |
|  |  |  | **(i) Did you find using this tool and answering its questions interesting?**  **(A) 4-level Likert-Scale.** | **(ii) Could you understand the questions asked by the tool?**  **(A) 4-level Likert-Scale.** | **(iii) Do you think that the tool could facilitate better treatment at the ED?**  **(A) 4-level Likert-Scale.** | **(iv) Did you feel better understood when speaking to the physician, because they were already aware of your medical problem?**  **(A) 4-level Likert-Scale.** | **(v) How do you rate the user experience provided to you in the tool (i.e. its usability)?**  **(B) 10-level Likert-Scale.** | **(vi) Would you recommend the app to others?**  **(B) 10-level Likert-Scale.** | **(i) Would the tool facilitate rapport with the patient?**  **(A) 4-level Likert-Scale.** | | **(ii) Would the tool provide medically helpful information?**  **(A) 4-level Likert-Scale.** | | **(iii) Would the tool (as currently implemented) save time?**  **(A) 4-level Likert-Scale.** | | **(iv) Would you recommend the tool to colleagues?**  **(B) 10-level Likert-Scale.** | | ***Degree of patient self-sufficiency.***  ***On the 4-level scale of assistance: 1 - high; 2 - medium; 3- low; 4 - none.*** |
|  |  |  | **Patient** | **Patient** | **Patient** | **Patient** | **Patient** | **Patient** | **Dr** | **Nurse** | **Dr** | **Nurse** | **Dr** | **Nurse** | **Dr** | **Nurse** | **Researcher** |
| 001 | Phase 1 | Internal Medicine | 3 | 4 | 1 | 2 | 9 | - | 1 | 4 | 1 | 3 | 1 | 3 | 1 | 8 | 4 |
| 002 | Phase 1 | Orthopaedics | 4 | 4 | 4 | 4 | 9 | 9 | 2 | - | 2 | - | 1 | - | 5 | - | 2 |
| 003 | Phase 1 | Surgery | 4 | 4 | 4 | 4 | 8 | 6 | 2 | - | 2 | - | 2 | - | 3 | - | 2 |
| 004 | Phase 1 | Orthopaedics | 3 | 4 | 3 | 3 | 8 | 10 | 3 | - | 4 | - | 3 | - | 7 | - | 2 |
| 005 | Phase 1 | Orthopaedics | 4 | 3 | 4 | 4 | 5 | 5 | 3 | - | 2 | - | 1 | - | 5 | - | 2 |
| 006 | Phase 1 | IM-Rheumatology | 4 | 4 | 4 | 4 | 7 | 7 | 4 | - | 3 | - | 3 | - | 7 | - | 1 |
| 007 | Phase 1 | Orthopaedics | 4 | 4 | 3 | 3 | 10 | 9 | 3 | - | 3 | - | 2 | - | 6 | - | 3 |
| 008 | Phase 1 | General Surgery | 4 | 4 | 3 | 3 | 8 | 10 | 3 | - | 3 | - | 3 | - | 5 | - | 3 |
| 009 | Phase 1 | Internal Medicine | 4 | 3 | 3 | 3 | 5 | 5 | 4 | - | 4 | - | 4 | - | 9 | - | 2 |
| 010 | Phase 1 | General Surgery | 3 | 3 | 2 | 3 | 7 | 7 | 4 | - | 4 | - | 4 | - | 9 | - | 3 |
| 011 | Phase 1 | General Surgery | 3 | 4 | 3 | 4 | 10 | 10 | 4 | - | 3 | - | 4 | - | 9 | - | 2 |
| 012 | Phase 1 | Orthopedics | 4 | 3 | 3 | 4 | 9 | 8 | 4 | - | 4 | - | 4 | - | 9 | - | 4 |
| 013 | Phase 1 | Psychiatry | 2 | 1 | 2 | 1 | 2 | 2 | 3 | - | 2 | - | 1 | - | 6 | - | 1 |
| 014 | Phase 1 | ENT | 3 | 4 | 1 | - | - | - | 4 | - | 4 | - | 4 | - | 10 | - | 3 |
| 015 | Phase 1 | Neurology | 4 | 2 | 3 | 3 | 8 | 5 | 4 | - | 4 | - | 4 | - | 9 | - | 3 |
| 016 | Phase 1 | Neurology | 2 | 1 | 1 | 2 | 4 | 3 | 1 | - | 1 | - | 1 | - | 2 | - | 3 |
| 017 | Phase 1 | Neurology | 3 | 2 | 3 | 2 | 6 | 6 | 3 | - | 3 | - | 2 | - | 7 | - | 1 |
| 018 | Phase 1 | Neurology | 3 | 3 | 2 | 2 | 1 | 4 | 3 | - | 2 | - | 2 | - | 5 | - | 1 |
| 019 | Phase 1 | IM-Nephrology | 2 | 2 | 2 | 2 | 3 | 3 | 3 | - | 3 | - | 2 | - | 6 | - | 1 |
| 020 | Phase 1 | Internal Medicine | 2 | 3 | 2 | 3 | 7 | 6 | 4 | - | 3 | - | 3 | - | 7 | - | 4 |
| 021 | Phase 1 | IM-Cardiology | 4 | 4 | 3 | 3 | 8 | 10 | 3 | - | 3 | - | 2 | - | 5 | - | 3 |
| 022 | Phase 1 | Dermatology | 2 | 1 | 1 | 2 | - | - | 2 | - | 2 | - | 2 | - | 4 | - | 2 |
| 023 | Phase 1 | Orthopaedics | 4 | 3 | 3 | 3 | 8 | 8 | 1 | - | 1 | - | 1 | - | 2 | - | 2 |
| 024 | Phase 1 | Orthopaedics | 4 | 4 | 4 | 4 | 10 | 6 | 1 | - | 1 | - | 1 | - | 2 | - | 3 |
| 025 | Phase 1 | IM-Gastroenterology | 2 | 2 | 3 | 3 | 8 | 8 | 3 | - | 3 | - | 2 | - | 5 | - | 1 |
| 026 | Phase 1 | Internal Medicine | 2 | 4 | 3 | 3 | 10 | 8 | 3 | - | 4 | - | 3 | - | 6 | - | 4 |
| 027 | Phase 1 | Internal Medicine | 3 | 3 | 4 | 4 | 6 | 5 | 3 | - | 2 | - | 2 | - | 4 | - | 3 |
| 028 | Phase 1 | Dermatology | 3 | 4 | 2 | 2 | 10 | - | 2 | - | 2 | - | 1 | - | 4 | - | 3 |
| 029 | Phase 1 | Neurology | 3 | 2 | 3 | 2 | 6 | 6 | 2 | - | 2 | - | 2 | - | 3 | - | 2 |
| 030 | Phase 1 | No diagnosis assigned | 4 | 4 | - | - | 6 | 5 | - | - | - | - | - | - | - | - | 3 |
| 031 | Phase 1 | Dermatology | 3 | 2 | 3 | 3 | 8 | 6 | 2 | - | 2 | - | 2 | - | 3 | - | 4 |
| 032 | Phase 1 | Neurology | 4 | 4 | 2 | 1 | 10 | 10 | 3 | - | 2 | - | 2 | - | 8 | - | 4 |
| 033 | Phase 1 | Dermatology | 4 | 4 | 2 | 2 | 8 | 6 | 3 | - | 2 | - | 1 | - | 4 | - | 4 |
| 034 | Phase 1 | Internal Medicine | 3 | 4 | - | - | 8 | 3 | - | - | - | - | - | - | - | - | - |
| 035 | Phase 1 | Neurology | 3 | 4 | - | - | 10 | 10 | 3 | - | 4 | - | 3 | - | 6 | - | 4 |
| 036 | Phase 1 | Neurology | 4 | 3 | 3 | 3 | 8 | 10 | 3 | - | 3 | - | 2 | - | 4 | - | 4 |
| 037 | Phase 1 | General Surgery | 4 | 3 | 4 | 4 | 8 | 9 | 3 | - | 2 | - | 2 | - | 6 | - | 4 |
| 038 | Phase 1 | Orthopaedics | 3 | 4 | 3 | 3 | 7 | 8 | 2 | - | 1 | - | 1 | - | 4 | - | 3 |
| 039 | Phase 1 | General Surgery | 3 | 2 | 3 | 3 | 8 | 8 | 4 | - | 2 | - | 3 | - | 6 | - | 4 |
| 040 | Phase 1 | Orthopaedics | 3 | 4 | 3 | 3 | 8 | 8 | 2 | - | 2 | - | 2 | - | 3 | - | 4 |
| 041 | Phase 1 | Dermatology | 4 | 4 | 1 | 1 | 10 | 10 | 2 | - | 2 | - | 2 | - | 3 | - | 3 |
| 042 | Phase 1 | Gynaecology | 3 | 3 | 3 | 3 | 6 | 6 | 3 | - | 3 | - | 3 | - | 5 | - | 2 |
| 043 | Phase 1 | Internal Medicine | 3 | 3 | 3 | 3 | 7 | 7 | 3 | - | 3 | - | 3 | - | 7 | - | 4 |
| 044 | Phase 1 | Internal Medicine | 3 | 3 | 2 | - | 5 | 9 | 3 | - | 3 | - | 3 | - | 6 | - | 3 |
| 045 | Phase 1 | Internal Medicine | 3 | 3 | 2 | 3 | 9 | 9 | 2 | - | 2 | - | 2 | - | 5 | - | 3 |
| 046 | Phase 1I | IM-Gastroenterology | 3 | 3 | 3 | 3 | 7 | 8 | 3 | - | 2 | - | 2 | - | 6 | - | 2 |
| 047 | Phase 1I | Internal Medicine | 4 | 4 | 4 | 4 | 9 | 9 | 3 | - | 2 | - | 2 | - | 5 | - | 3 |
| 048 | Phase 1I | IM-Cardiology | 4 | 4 | 4 | 4 | 2 | 2 | 3 | - | 2 | - | 2 | - | 7 | - | 3 |
| 049 | Phase 1I | Neurology | 4 | 4 | 4 | 3 | 7 | 10 | 3 | 4 | 3 | 4 | 2 | 2 | 6 | 6 | 4 |
| 050 | Phase 1I | General Surgery | 4 | 4 | 3 | 4 | 9 | 10 | 4 | 4 | 4 | 4 | 1 | 4 | 8 | 8 | 4 |
| 051 | Phase 1I | General Surgery | 4 | 4 | - | - | 9 | 9 | - | 3 | - | 3 | - | 4 | - | 7 | 4 |
| 052 | Phase 1I | Neurology | 4 | 2 | 1 | 1 | 9 | 3 | 3 | 4 | 4 | 4 | 3 | 3 | 7 | 9 | 4 |
| 053 | Phase 1I | Internal Medicine | 4 | 3 | 3 | 4 | 1 | 1 | 3 | 3 | 3 | 3 | 2 | 2 | 7 | 8 | 3 |
| 054 | Phase 1I | Neurology | 4 | 3 | 4 | 3 | 6 | 5 | 3 | 3 | 3 | 3 | 2 | 3 | 5 | 8 | 3 |
| 055 | Phase 1I | IM-Gastroenterology | 3 | 4 | 4 | 4 | 4 | 5 | 3 | 4 | 3 | 3 | 3 | 4 | 7 | 8 | 4 |
| 056 | Phase 1I | Neurology | 4 | 3 | 3 | 3 | 7 | 5 | 3 | 4 | 3 | 3 | 3 | 3 | 7 | 8 | 4 |
| 057 | Phase 1I | Orthopaedics | 4 | 3 | - | - | 9 | 10 | 2 | 4 | 2 | 4 | 2 | 4 | 5 | 8 | 4 |
| 058 | Phase 1I | Internal Medicine | 4 | 4 | 4 | 4 | 8 | 8 | 4 | - | 3 | - | 2 | - | 7 | - | 3 |
| 059 | Phase 1I | Internal Medicine | 4 | 4 | 2 | 2 | 8 | 10 | 2 | - | 2 | - | 2 | - | 7 | - | 3 |
| 060 | Phase 1I | General Surgery | 3 | 3 | 3 | 3 | 8 | 7 | 3 | - | 3 | - | 2 | - | 5 | - | 3 |
| 061 | Phase 1I | Neurology | 3 | 4 | - | - | 8 | 8 | 1 | - | 3 | - | 3 | - | 7 | - | 3 |
| 062 | Phase 1I | Orthopaedics | 4 | 4 | 1 | 1 | 10 | 10 | 3 | - | 3 | - | 2 | - | 6 | - | 4 |
| 063 | Phase 1I | IM-Oncology | 4 | 4 | 4 | 4 | 8 | 8 | 3 | - | 3 | - | 1 | - | 7 | - | 1 |
| 064 | Phase 1I | Neurology | 4 | 3 | 2 | 1 | 2 | 7 | 3 | - | 3 | - | 2 | - | 5 | - | 4 |
| 065 | Phase 1I | Neurology | 4 | 4 | 4 | 4 | 8 | 8 | 3 | - | 2 | - | 1 | - | 5 | - | 3 |
| 066 | Phase 1I | Orthopaxedics | 3 | 3 | 3 | 3 | 7 | 8 | 4 | - | 4 | - | 3 | - | 8 | - | 4 |
| 067 | Phase 1I | IM-Cardiology | 4 | 4 | 1 | 1 | 7 | 7 | 3 | - | 2 | - | 2 | - | 6 | - | 4 |
| 068 | Phase 1I | Internal Medicine | 3 | 3 | 3 | 3 | 6 | 7 | 2 | - | 3 | - | 3 | - | 6 | - | 4 |
| 069 | Phase 1I | Internal Medicine | 4 | 4 | 4 | 4 | 10 | 10 | 3 | - | 3 | - | 3 | - | 5 | - | 4 |
| 070 | Phase 1I | Internal Medicine | 4 | 4 | 2 | 3 | 7 | 7 | 1 | - | 1 | - | 1 | - | 10 | - | 4 |
| 071 | Phase 1I | General Surgery | 4 | 3 | 2 | 3 | 9 | 8 | 4 | - | 4 | - | 4 | - | 3 | - | 4 |
| 072 | Phase 1I | Internal Medicine | 3 | 4 | 4 | 3 | 8 | 7 | 3 | - | 3 | - | 3 | - | 8 | - | 3 |
| 073 | Phase 1I | Neurology | 4 | 4 | 4 | 4 | 9 | 9 | 2 | - | 2 | - | 2 | - | 3 | - | 2 |
| 074 | Phase 1I | Neurology | 4 | 4 | 4 | 4 | 9 | 9 | 3 | - | 3 | - | 2 | - | 6 | - | 3 |
| 075 | Phase 1I | IM-Cardiology | 2 | 4 | 2 | 3 | 3 | 3 | 3 | - | 3 | - | 2 | - | 5 | - | 4 |
| 076 | Phase 1I | Neurology | 4 | 3 | 4 | 4 | 2 | 9 | 2 | - | 2 | - | 1 | - | 5 | - | 4 |
| 077 | Phase 1I | ENT | 3 | 3 | 3 | 4 | 7 | 7 | 3 | - | 2 | - | 1 | - | 5 | - | 4 |
| 078 | Phase 1I | IM-Gastroenterology | 4 | 4 | 4 | 4 | 10 | 10 | 3 | - | 3 | - | 3 | - | 7 | - | 3 |
| 079 | Phase 1I | ENT | 4 | 4 | 3 | 2 | 10 | 10 | 3 | - | 1 | - | 1 | - | 5 | - | 4 |
| 080 | Phase 1I | Neurology | 4 | 4 | 2 | 3 | 7 | 7 | 3 | - | 2 | - | 2 | - | 6 | - | 4 |
| 081 | Phase 1I | Neurology | 3 | 3 | 3 | 3 | 8 | 8 | 4 | - | 3 | - | 4 | - | - | - | 3 |
